# Three-Dimensional Object Motion and Velocity Estimation Using a Single Computational RGB-D Camera

**DOI:** 10.3390/s150100995

**Published:** 2015-01-08

**Authors:** Seungwon Lee, Kyungwon Jeong, Jinho Park, Joonki Paik

**Affiliations:** Image Processing and Intelligent Systems Laboratory Graduate School of Advanced Imaging Science, Multimedia, and Film Chung-Ang University, Seoul 156-756, Korea; E-Mails: superlsw@gmail.com (S.L.); fjqmsa@gmail.com (K.J.); dkskzmffps@gmail.com (J.P.)

**Keywords:** object tracking, depth estimation, computational camera, image registration, 3D image acquisition

## Abstract

In this paper, a three-dimensional (3D) object moving direction and velocity estimation method is presented using a dual off-axis color-filtered aperture (DCA)-based computational camera. Conventional object tracking methods provided only two-dimensional (2D) states of an object in the image for the target representation. The proposed method estimates depth information in the object region from a single DCA camera that transforms 2D spatial information into 3D model parameters of the object. We also present a calibration method of the DCA camera to estimate the entire set of camera parameters for a practical implementation. Experimental results show that the proposed DCA-based color and depth (RGB-D) camera can calculate the 3D object moving direction and velocity of a randomly moving object in a single-camera framework.

## 1. Introduction

Object tracking is a very popular research topic in the computer vision field because of its wide applications in video surveillance systems, intelligent driver assistant systems, robot vision, etc. [1,2]. Conventional object tracking methods provided only two-dimensional (2D) states of an object in the image and therefore cannot deal with the occlusion and depth-related problems. For solving these problems, estimation of three-dimensional (3D) depth information has been intensively studied for the past several decades [3–6].

The traditional approach to 3D image acquisition or depth estimation involves the use of two cameras that capture two images of the same scene from different viewpoints [7,8]. Despite many advantages, the stereo vision system has a fundamental limitation that highly accurate calibration is required for both cameras. In addition, stereo cameras require both calibration and rectification steps for the alignment of two cameras. However, even after completion of the two steps, the rectification step should be performed again, if the stereo camera is influenced by various external factors that break the alignment between two cameras. As an alternative approach, Panasonic's LUMIX G 12.5 mm F12 lens can acquire two images with different viewpoints by dividing the imaging sensor into two regions. Although this system mimics the stereo vision in a single-camera framework, the reduced resolution is the fundamental disadvantage.

Another single camera-based depth estimation approach is the pupil plane coding method that modifies an aperture of a lens for coding geometric depth information into the image [9]. Recently, three apertures covered with red, green and blue filters have been used for the same purpose [10], where three color-filtered apertures (TCA) generate color shifts among channels depending on the distance of an object. Kim *et al.* proposed a multifocusing image restoration algorithm using the distance estimation in the color shifted image using the TCA [11]. Lee *et al.* proposed a simultaneous object tracking and depth estimation system using the TCA camera [12]. However, these TCA-based methods produce redundant pairs of disparity vectors and do not establish the theoretically complete relationship between color shifting and the real distance. Lee *et al.* proposed a novel configuration of dual off-axis color-filtered apertures (DCA) to remove the disparity redundancy of the color shifting vectors and to increase the size of an individual aperture to receive more incoming light [13]. The mathematical model of the DCA configuration with the relationship between color shifting values and the actual distance of the object were proposed in [14].

In this paper, we present a novel 3D object moving direction and velocity estimation method for robust object tracking using a DCA-based computational RGB-D camera. The incremental learning-based approach [15] is used for object tracking from the DCA camera, and the color shifting value (CSV), which is related to the distance of the object, is estimated in the region of the tracked object. The states of the object at the image coordinate are then transformed into the 3D parameters of the object using the estimated CSV. Finally, the direction and velocity of a moving 3D object are simultaneously calculated while tracking the object. We also present a calibration method of the DCA camera to estimate the entire set of camera parameters for real applications. Although this work shares the concept of multiple, color-filtered apertures in the previous work [11–14], the original contribution includes: (i) the incremental learning-based object tracking algorithm that is optimized for the DCA camera system; (ii) single camera-based simultaneous 3D object moving direction and velocity estimation; and (iii) a novel calibration method that can be used for a DCA camera system.

The paper is organized as follows. Section 2 presents the background of the DCA camera, and Sections 3 and 4 describe the object moving direction and velocity estimation for tracking and the DCA camera calibration, respectively. Experimental results are provided in Section 5, and Section 6 concludes the paper.

## 2. Dual Off-Axis Color-Filtered Aperture-Based Camera

**Figure 1.**
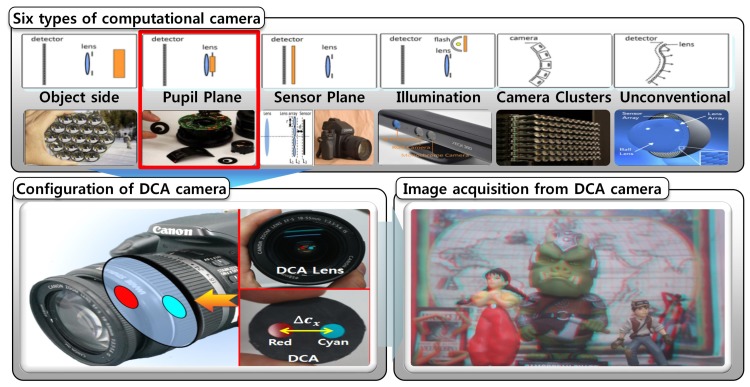
Six types of computational cameras and the proposed dual off-axis color-filtered aperture (DCA) camera [9].

Recently, computational imaging systems have been widely used for obtaining additional information, while traditional imaging systems only acquire intensity and color information. A computational imaging system projects rays, which is altered by specially-designed optics, in the light field of the scene onto the image sensor using novel optics and the correspondingly developed image processing algorithms. These systems can produce a new type of image that is potentially useful for re-focusing, scene segmentation and 3D computer vision. Computational imaging systems can be broadly classified into six categories, as shown in Figure 1 [9].

The pupil plane coding places a specially designed pattern at the aperture in front of the lens to easily configure the computational camera for depth information. By incorporating the concept of pupil plane coding, we design a novel computational imaging system by simply inserting an appropriately resized DCA into any general optical system in the single-camera framework.

The aperture of an optical system is the opening device that adjusts the amount of light entering the image sensor. The center of the aperture in the traditional imaging system is generally aligned with the optical axis of the lens. If there are two off-axis apertures, the convergence pattern of the projected point on the image plane is divided into the two projected points if the object is not located at the plane of focus. The distance between two separated convergence patterns depends on the distance of the object from the camera. It is noted that we cannot estimate the distance between two projected regions if they are mixed together.

**Figure 2.**
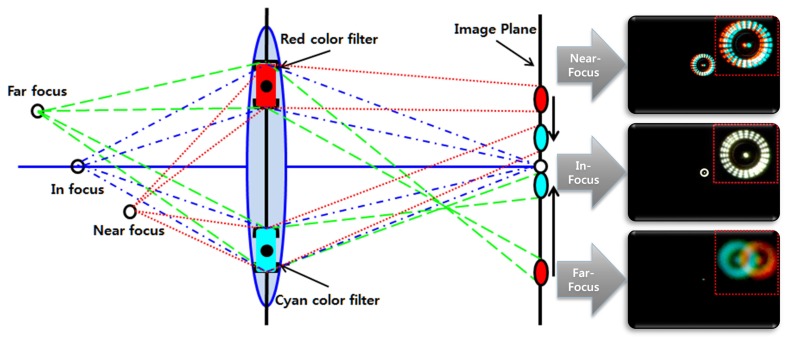
The convergence patterns of objects at three different distances in the DCA configuration.

In order to separate two regions, we covered apertures using two different color filters that generate geometric disparity between two color images. For the complete color shift model using the DCA camera, we use two apertures with red (R) and cyan (C) filters, whose centers are located on the same line crossing the optical axis. The color shift model can provide the geometric disparity of misalignment, which can be estimated from the amount of color deviation between the pair of two projected points, as shown in Figure 2. Thus, the distance of the object can be estimated using the color shifting value (CSV) that corresponds to the length of the amount of misalignment between color channels from the color-misaligned image.

## 3. Object Moving Direction and Velocity Estimation with Tracking

The object region should be continuously tracked to estimate the 3D motion of the object while the position and scale of the object change. We use the incremental learning-based method for robust object tracking [15].

The statistical object tracking problem is usually defined as the Bayesian inference with a hidden Markov model. Given the state of an object at time *t*, denoted as **o***_t_*, cumulated observations up to time *t*, denoted as *Y_1_*_:_*_t_*, the Bayesian filter updates *a posteriori* probability *p*(**o***_t_*|**y**_1:_*_t_*) with the following rule,

(1) $$p({\text{o}}_{t}|{\text{y}}_{1:t})\propto p({\text{y}}_{t}|{\text{o}}_{t})\int p({\text{o}}_{t}|{\text{o}}_{t-1})p({\text{o}}_{t-1}|{\text{y}}_{1:t-1})d{\text{o}}_{t-1}$$

where *p*(**y***_t_*|**o***_t_*) represents the observation model as a likelihood term that measures the similarity between the observation at the estimated state and the given model and *p*(**o***_t_*|**o***_t_*_−1_) the transition model as a prior term, which predicts the next state **o***_t_* based on the previous state **o***_t_*_−1_. With the posterior probability *p*(**o***_t_*|**y**_1:_*_t_*), we obtain the maximum *a posteriori* (MAP) estimate over *N* samples at time *t* as:

(2) $${\text{o}}_{t}^{\text{MAP}}=\underset{{\text{o}}_{t}^{n}}{\arg\max}p({\text{o}}_{t}^{n}|{\text{y}}_{1:t}),\text{for}n=1,\ldots ‥,N$$

where ${\text{o}}_{t}^{\text{MAP}}$ denotes the best configuration, which can explain the current state with the given model. The observation model is updated by the incremental principle components analysis (PCA) algorithm proposed by Ross, and then, the current state ${\text{o}}_{t}^{\text{MAP}}$ is determined by the distance metric in this work [15]. The transition model is formulated by a random walk with a Gaussian distribution.

Let the 2D state of the object **x***_t_* at time *t* in the image coordinates denote **x**_t_ = {*x_t_*, *y_t_*, *w_t_*, *h_t_*}, where *x_t_* and *y_t_* indicate the center position of the object, *w_t_* and *h_t_* represent the width and the height of the object, respectively. In order to convert the image coordinate system into the 3D camera coordinate system, we need to know the distance *Z* of the object and camera intrinsic parameters, including the focal lengths *f_x_*, *f_y_* and the principal point *s_x_*, *s_y_*.

Because two disparities between red and green and red and blue are the same with respect to the horizontal direction, the distance measure combines two energy functions as:

(3) $$\begin{array}{ll} E(\Delta x) & =\sum_{x,y\in \Omega }{\left(I^{r}(x,y)-I^{g}(x+\Delta x,y)\right)}^{2}\\ & +\sum_{x,y\in \Omega }{\left(I^{r}(x,y)-I^{b}(x+\Delta x,y)\right)}^{2} \end{array}$$

where Δ*x* represents the CSV, Ω the extracted object region and *I* the image acquired by the DCA camera. Superscripts *r*, *g* and *b* respectively represent the red, green and blue color channels. Since this error function is nonlinear, it cannot be analytically minimized. To simplify the minimization, we approximate this error function using a first-order truncated Taylor series expansion [16] as:

(4) $$\begin{array}{ll} E(\Delta x) & =\sum_{x,y\in \Omega }{\left(I_{t}^{rg}(x,y)-\Delta xI_{t}^{rg}(x,y)\right)}^{2}\\ & +\sum_{x,y\in \Omega }{\left(I_{t}^{rb}(x,y)-\Delta xI_{x}^{rb}(x,y)\right)}^{2} \end{array}$$

where $I_{t}^{rc}=I^{r}-I^{c}$, for *c* ∈ {*g*, *b*}, and $I_{x}^{rc}$ represents the first derivative of (*I^r^* + *I^c^*)/2 using the Sobel operator in the horizontal direction. Since *E*(Δ*x*) is a quadratic function of Δ*x*, a closed-from solution for minimizing the energy can be found by differentiation with respect to Δ*x* and setting the result equal to zero as:

(5) $$\Delta x=\sum_{x,y\in \Omega }\frac{\left[I_{x}^{rg}(x,y)I_{t}^{rg}(x,y)+I_{x}^{rb}(x,y)I_{t}^{rb}(x,y)\right]}{\left[{\left(I_{x}^{rg}(x,y)\right)}^{2}+{\left(I_{x}^{rb}(x,y)\right)}^{2}\right]}$$

A more accurate estimation of Δ*x* can be performed by a Gaussian pyramid-based iterative coarse-to-fine approach for accommodating large color shifting values between color channels [17]. In [13], Lee *et al.* estimated the dense depth map in the entire image. However, the proposed method estimates only one CSV by minimizing the error function in the object region. If the object region is large enough to contain meaningful features, it is almost always reliable.

**Figure 3.**
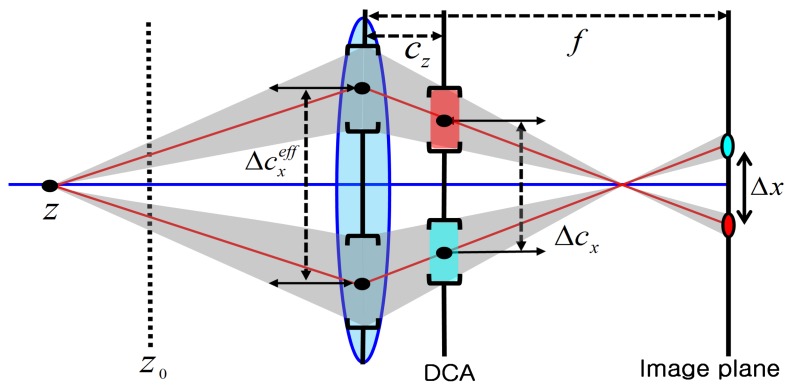
The configuration of the DCA camera.

In [14], the relation between Δ*x* and the object distance *Z* has been derived as:

(6) $$\Delta x=f^{2}\frac{Z_{0}-Z}{\left(Z_{0}-f)(Z\cdot f-(Z-f)c_{z}\right)}\Delta c_{x}$$

where *f* represents the focal length, Δ*c_x_* the distance between the two off-axis apertures, *c_z_* the distance of the DCA away from the lens and *Z*_0_ the plane of focus. Figure 3 shows the configuration of the DCA camera. If we assume that both *Z* and *Z*_0_ are sufficiently larger than the focal length, then the movement of the projection is approximately computed as:

(7) $$\Delta x\approx f\left(\frac{1}{Z}-\frac{1}{Z_{0}}\right)\frac{f}{f-c_{z}}\Delta c_{x}$$

where *f*/(*f* − *c_z_*) determines the distance between the two equivalent apertures, $\Delta c_{x}^{\text{eff}}=(f/(f-c_{z}))\cdot \Delta c_{x}$. If *c_z_* is zero, $\Delta c_{x}^{\text{eff}}$ is equal to Δ*c_x_*.

If the parameters in Equation (7) are expressed in millimeters, then Δ*x* is also determined in millimeters. In order to express Δ*x* in pixels, Δ*x* has to be multiplied by the distance *α_x_* between two pixels, which is given as:

(8) $$\alpha_{x}=S_{h}/M_{h}$$

where *S_h_* represents the size of an image sensor with respect to the horizontal direction and *M_h_* the size of the image resolution with respect to the horizontal. *f* is redefined as *f_x_ = f*/*α_x_*, and then, Equation (8) can be rewritten as:

(9) $$\Delta x\approx f_{x}\frac{\Delta c_{x}^{\text{eff}}}{Z}-f_{x}\frac{\Delta c_{x}^{\text{eff}}}{Z_{0}}$$

Equation (9) is similar to the relationship between the disparity and the depth in the stereo vision defined as $\Delta D=f_{x}\frac{B}{Z}$, where *B* represents the baseline, except theoffset given as $f\frac{\Delta c_{x}^{\text{eff}}}{z_{0}}$. Solving (9) for *Z* yields:

(10) $$Z=\frac{f_{x}Z_{0}\Delta c_{x}^{\text{eff}}}{f_{x}\Delta c_{x}^{\text{eff}}+Z_{0}\Delta x}$$

Given *Z*, the 2D states of the object *x_t_* and *y_t_* are transformed into 3D camera coordinate as:

(11) $$X_{t}=(x_{t}-s_{x})\cdot Z_{t}/f_{x}\;\text{and}\;Y_{t}=(y_{t}-s_{y})\cdot Z_{t}/f_{y}$$

where *s_x_* and *s_y_* are the principal points with respect to horizontal and vertical directions, respectively.

The 2D states of the object *w_t_* and *h_t_* are transformed into 3D camera coordinates as:

(12) $$W=w\cdot Z/f_{x}\;\text{and}\;H=h\cdot Z/f_{y},$$

3D object moving direction **D** and velocity *V* are then calculated as:

(13) $$D=\{\dot{X}=X_{t}-X_{t-k},\dot{Y}=Y_{t}-Y_{t-k},\dot{Z}=Z_{t}-Z_{t-k}\}$$

(14) $$V=\sqrt{{\dot{X}}^{2}+{\dot{Y}}^{2}+{\dot{Z}}^{2}}$$

where *Ẋ*, *Ẏ* and *Ż* represent the amount of the object moving with respect to the *x*-, *y*- and *z*-coordinates, respectively, *k* represents the number of frames per second. Finally, the proposed 3D state of the object is defined as **ȯ***_t_* = {**o***_t_*, **O***_t_*, **D***_t_*, *V_t_*}, where **O***_t_* = {*X_t_*, *Y_t_*, *Z_t_*}.

In **ȯ***_t_*, the state of the object to be used for object tracking is **o***_t_*. After determining ${\text{o}}_{t}^{\text{MAP}}$, the final 3D state of the object **ȯ***_t_* is then computed. Continuously estimated *Z* may have a minor oscillation due to the estimation error of the CSV. For robust estimation of *Z*, we use Kalman filtering to predict and compensate temporally changing CSV.

## 4. DCA Camera Calibration

For accurate depth estimation, the entire set of camera parameters, except Δ*x*, should be calibrated. Calibration of the DCA camera consists of two steps: (i) estimation of the camera parameters, including the focal lengths, principal point and lens distortion coefficients; and (ii) estimation of the DCA parameters, including *Z*_0_ and *c_z_*.

**Figure 4.**
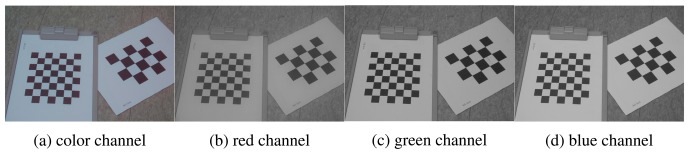
Calibration patterns for estimating the camera parameters, (**a**) Color channel; (**b**) Red channel; (**c**) Green channel; (**d**) Blue channel.

Although each color channel has the same lens distortion coefficients, because the DCA camera uses a single lens, *s_x_* and *s_y_* have to be estimated in each color channel due to two off-axis apertures, as shown Figure 4. For the calibration of the camera parameters, we took the images using a checkerboard at different camera locations and used a calibration tool at each color channel.

**Figure 5.**
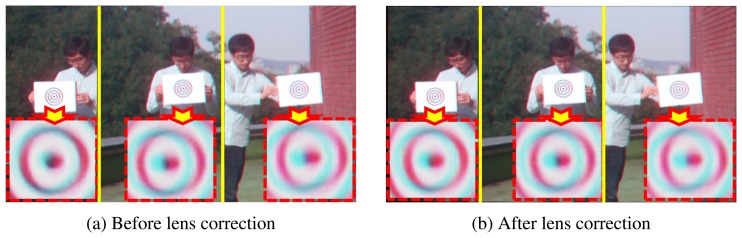
The result of the lens distortion correction. (**a**) Before lens correction; (**b**) After lens correction.

Although the object is located at the same distance, each object has different CSVs before lens correction, as shown in Figure 5a. However, after lens correction, the objects located at the same distance also have the same CSVs, as shown in Figure 5b.

**Figure 6.**
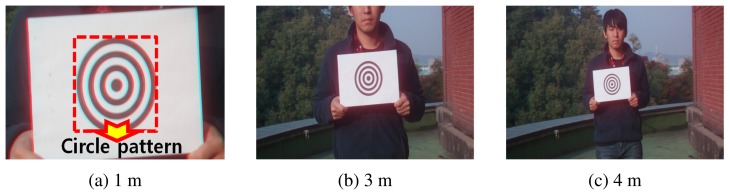
A concentric circle pattern for calibrating DCA parameters. (**a**) 1 m; (**b**) 3 m; (**c**) 4 m.

For solving *Z* as in Equation (10) from Δ*x*, the unknown parameters *Z*_0_ and *c_z_* should be determined. We use a concentric circle pattern for calibrating the DCA camera and then take the image of the pattern at various distances, as shown in Figure 6. Because we know the real diameter of the circle, the distance of the concentric circle pattern can be calculated as:

(15) $$Z=f_{x}\cdot \frac{W}{w}$$

where *W* represents the real diameter of the circle in meters and *w* represents the diameter of the circle in pixels. Rearranging Equation (10) yields:

(16) $$Z\;\cdot \;Z_{0}^{-1}-\frac{\Delta x\cdot Z\cdot \alpha_{x}}{c_{x}\cdot f^{2}}c_{z}=1-\frac{\Delta x\cdot Z\cdot \alpha_{x}}{c_{x}\cdot f}$$

For solving this equation, we need to develop more than two linear equations. Therefore, we estimate Δ*x* from the circle pattern of more than two images and then calculate *Z* using Equation (15). Using these values, Equation (16) can be developed as a matrix form as:

(17) $$\left[\begin{array}{cc} Z_{1} & -\frac{\Delta x_{1}\cdot Z_{1}\cdot \alpha }{c_{x}\cdot f^{2}}\\ Z_{2} & -\frac{\Delta x_{2}\cdot Z_{2}\cdot \alpha }{c_{x}\cdot f^{2}}\\ \vdots & \vdots \\ Z_{n} & -\frac{\Delta x_{n}\cdot Z_{n}\cdot \alpha }{c_{x}\cdot f^{2}} \end{array}\right]\;\left[\begin{array}{c} Z_{0}^{-1}\\ c_{z} \end{array}\right]=\left[\begin{array}{c} 1-\frac{\Delta x_{1}\cdot Z_{1}\cdot \alpha }{c_{x}\cdot f}\\ 1-\frac{\Delta x_{2}\cdot Z_{2}\cdot \alpha }{c_{x}\cdot f}\\ \vdots \\ 1-\frac{\Delta x_{n}\cdot Z_{n}\cdot \alpha }{c_{x}\cdot f} \end{array}\right]$$

where *Z_n_* and Δ*x_n_* represent the *n*-th object distance and the corresponding CSV, respectively. Finally, *Z*_0_ and *c_z_* can be solved by multiplying the pseudo inverse matrix. As a result of the calibrations of *Z*_0_ and *c_z_*, we are able to calculate the distance of the object from the estimated color shifting value, Δ*x*, between color channels in the image using Equation (10).

In [14], Lee *et al.* estimated *c_z_* by assuming that two objects are placed at *Z*_0_ and *Z*_1_, which should be given before the estimation process. It also needs manual identification of whether the object is placed at the in-focus position to determine *Z*_0_. However, the proposed calibration method estimates the unknown parameters *Z*_0_ and *c_z_* using the least squares optimization method with the distance data acquired at different locations using the known size of the circle pattern. Because the proposed method just takes the image using the circle pattern and does not need to measure the distance of the object, it can better estimate the calibration parameters than [14].

## 5. Experimental Results

To demonstrate the feasibility of the DCA-based RGB-D camera for the distance estimation of the tracked object, we used a Sony NEX6 digital single lens reflected (DSLR) camera with a 18 − 55 mm lens. The resolution of the video sequence is 1920 × 1080. The DCA lens is configured by the red and the cyan color filters, and the distance between the two apertures Δ*c_x_* is set to 6 mm.

**Figure 7.**
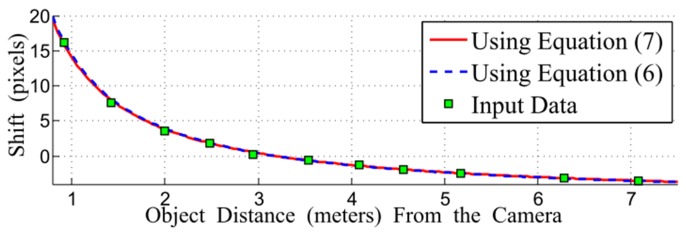
Plot of Δ*x versus* the distance *Z* of an object from the camera (*f* = 49.8 mm, *z*_0_ = 3250 mm, *c_z_* = −10 mm).

Figure 7 shows comparison of the input data for the DCA calibration. As shown in the figure, the curve of the function using Equation (9) closely passes through the input data. In addition, Equation (7) is almost the same as its approximated version in (9).

**Figure 8.**
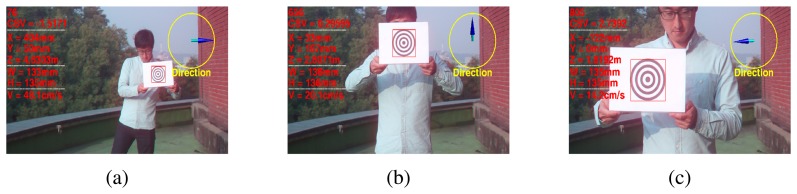
Estimation results of the 3D states of the object **xẋ***_t_* using the proposed method. (**a**) 76th frame; (**b**) 656th frame; (**c**) 805th frame.

Figure 8 shows the results of the proposed 3D state estimation of the tracked object. The images were taken of this object at distances that ranged from 5 m to 1 m, and cross pattern was moved at 1-m intervals.

**Figure 9.**
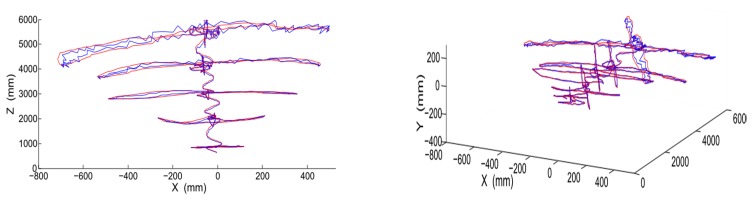
3D trajectory of the moving object (red, with Kalman filtering; blue, without Kalman filtering).

Figure 9 shows the 3D trajectory of the moving object in the camera coordinate system. The trajectory without Kalman filtering has minor oscillations due to the estimation error. However, the trajectory with Kalman filtering smoothly changes without oscillation.

**Figure 10.**
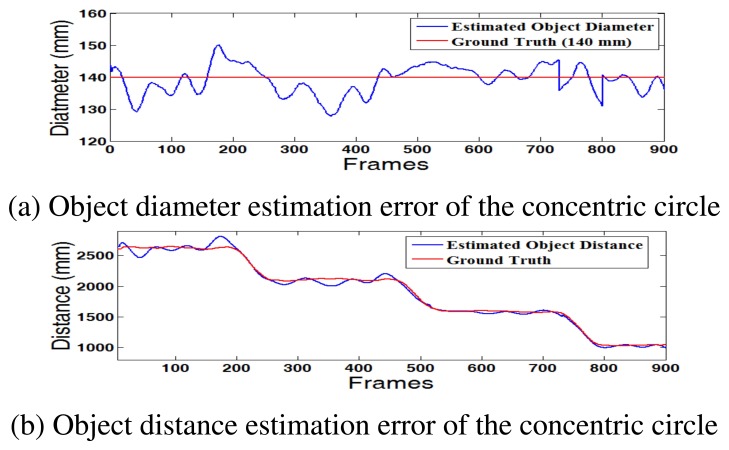
Comparison of the object diameter and distance estimation with the ground truth. (**a**) Object diameter estimation error of the concentric circle; (**b**) Object distance estimation error of the concentric circle.

Figure 10 shows the estimation error of the distances and diameters of the concentric circle. This result can provide a key indicator for accuracy of the depth estimation. If the estimated diameter is similar to the real value, 140 mm, the estimated 3D distance is also accurate. As shown in Figure 10a, the proposed method robustly estimates the diameter of the circle with an error less than ±10 mm.

**Figure 11.**
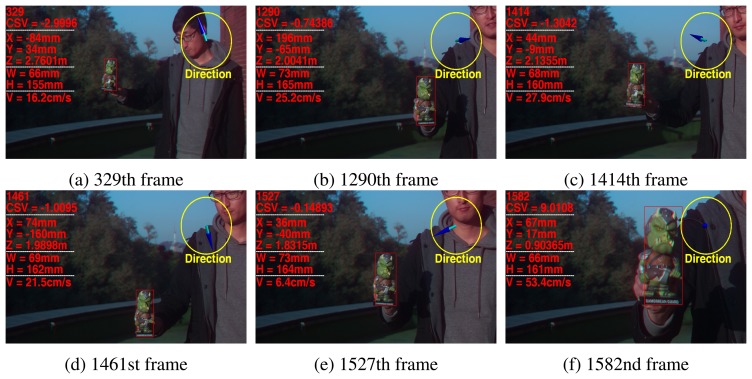
Estimation results of the proposed 3D states of the object **xẋ***_t_* using the proposed method. (**a**) 329th frame; (**b**) 1290th frame; (**c**) 1414th frame; (**d**) 1461st frame; (**e**) 1527th frame; (**f**) 1582nd frame.

Figure 11 shows the results of the estimated 3D states of the tracked object in another scene. The object having complex patterns was also successfully tracked with the proposed 3D states of the object.

As a result, the proposed method can estimate the 3D states of an object with a single object motion. However, it cannot accurately estimate the 3D states of multiple moving objects with possible occlusions or fade-in/-out, because the object tracking method may fail to track the object.

## 6. Conclusions

In this paper, we proposed a novel 3D object direction and velocity estimation method for object tracking using the DCA-based computational RGB-D camera. We estimated the amount of the color shifting value as the disparity by minimizing the error function. After the proposed DCA camera calibration, the 2D states of the object were converted into the 3D camera coordinates using an approximated mathematical model of the relationship between color shifting values and the actual distance of the object. Finally, the 3D object moving direction and velocity are calculated by the temporal changes of the object.

Based on the experimental results, the DCA camera-based object tracking system can successfully estimate the three-dimensional direction and velocity of a randomly-moving object. More accurate depth estimation with the extended range will be possible using an improved sub-pixel interpolation-based registration method in future research.

## Supplementary Materials

Supplementary materials can be accessed at: http://www.mdpi.com/1424-8220/15/1/995/s1.

## References

[1] Gade R., Moeslund T. (2014). Thermal tracking of sports players. Sensors.

[2] Li X., Guo R., Chen C. (2014). Robust pedestrian tracking and recognition from FLIR video a unified approach via sparse coding. Sensors.

[3] Le A., Jung S., Won C. (2014). Directional joint bilateral filter for depth images. Sensors.

[4] Fernandez-Sanchez E., Diaz J., Ros E. (2013). Background subtraction based on color and depth using active sensors. Sensors.

[5] Blanco C., Mantecon T., Camplani M., Jaureguizar F., Salgado L., Carcia N. (2014). Foreground segmentation in depth imagery using depth and spatial dynamic models for video surveillance applications. Sensors.

[6] Zhu Y., Fujimura K. (2010). A bayesian framework for human body pose tracking from depth image sequences. Sensors.

[7] Dhond U., Aggarwal J. (1989). Structure from stereo-a review. IEEE Trans. Syst. Man Cybern..

[8] Schastein D., Szeliski R. (2012). A taxonomy and evaluation of dense two-frame stereo correspondence algorithm. Int. J. Comput. Vision.

[9] Zhou C., Nayar S. (2011). Computational cameras: Convergence of optics and processing. IEEE Trans. Image Process..

[10] Maik V., Cho D., Shin J., Har D., Paik J. (2007). Color shift model-based segmentation and fusion for digital autofocusing. J. Imaging Sci. Technol..

[11] Kim S., Lee E., Hayes M., Paik J. (2012). Multifocusing and depth estimation using a color shift model-based computational camera. IEEE Trans. Image Process..

[12] Lee S., Lee J., Paik J. Simultaneous Object Tracking and Depth Estimation Using Color Shifting Property of a Multiple Color-Filter Aperture Camera.

[13] Lee S., Kim N., Jung K., Hayes M., Paik J. Single Image-Based depth Estimation Using Dual off-Axis Color Filtered Aperture Camera.

[14] Lee S., Hayes M., Paik J. (2013). Distance estimation using a single computational camera with dual off-axis color filtered apertures. Opt. Express.

[15] Ross D., Lim J., Lin R., Yang M. (2008). Incremental learning for robust visual tracking. Int. J. Comput. Vision.

[16] Lucas B., Kanade T. An Iterative Image Registration Technique with an Application to Stereo Vision.

[17] Periaswamy S., Farid H. (2003). Elastic registration in the presence of intensity variations. IEEE Trans. Med. Imaging.

